# Cerebellar MRI-based radiomics models for identifying mild cognitive impairment: a retrospective multicenter study in Southeast China

**DOI:** 10.3389/fnagi.2025.1566247

**Published:** 2025-07-11

**Authors:** Jianping Lu, Guoen Cai, Naian Xiao, Kunmu Zheng, Qinyong Ye, Xiaochun Chen

**Affiliations:** ^1^Department of Neurology, Fujian Medical University Union Hospital, Fuzhou, China; ^2^Institute of Clinical Neurology, Fujian Medical University, Fuzhou, China; ^3^Fujian Key Laboratory of Molecular Neurology and Institute of Neuroscience, Fujian Medical University, Fuzhou, China; ^4^Department of Neurology, The Third Hospital of Xiamen University, Fuzhou, China; ^5^Department of Neurology, The First Affiliated Hospital of Xiamen University, Fuzhou, China

**Keywords:** cognitive dysfunction, mild cognitive impairment, Alzheimer's disease, cerebellum, magnetic resonance imaging, radiomics

## Abstract

**Objective:**

This study aimed to investigate the role of cerebellar magnetic resonance imaging (MRI) features in identifying mild cognitive impairment (MCI).

**Methods:**

This retrospective multicenter study included patients with MCI, patients with Alzheimer's disease (AD), and healthy controls (HCs) from three tertiary hospitals in China (January 2022–December 2023). Cerebellar and hippocampal radiomics features were extracted from T1-, T2-, and T2-FLAIR-weighted MRI. A sparse representation classifier was developed using 10-fold cross-validation and was validated on independent datasets. Diagnostic performance was assessed through sensitivity, specificity, and ROC-AUC values.

**Results:**

A total of 87 patients with MCI, 109 patients with AD, and 55 healthy controls (HCs) matched by gender and age were included for model construction and validation. Additionally, 13 patients with MCI and 26 patients with AD were included for external validation. The 10-fold cross-validation accuracy and ROC AUC for identifying cognitive impairment (CI) in the training set using a combination of cerebellar T1, T2, and T2-FLAIR weighted images were better than those of hippocampal models (91.0% vs. 86.8%, 0.943 vs. 0.931). The accuracy and ROC AUC in the independent test set were similar (89.3% vs. 89.3%, 0.908 vs. 0.906). The 10-fold cross-validation accuracy and ROC AUC for identifying MCI in the training set, using a combination of cerebellar T1, T2, and T2-FLAIR weighted images, were similar to those of hippocampal models (85.2% vs. 83.7%, 0.877 vs. 0.905). Furthermore, the results were consistent with the external validation set (89.7% vs. 93.1%, 0.962 vs. 0.974).

**Conclusion:**

Cerebellar MRI radiomics models exhibit diagnostic accuracy comparable to hippocampal models for identifying CI and MCI, supporting the cerebellum's role in detecting early cognitive dysfunction. These findings provide novel insights into cerebellar contributions to AD pathophysiology and offer potential biomarkers for clinical application.

## Introduction

Alzheimer's disease (AD) is a neurodegenerative disease characterized by varying degrees of cognitive impairment (CI). The primary clinical manifestations of AD include progressive memory loss, aphasia, decline in executive functions, and psychiatric symptoms including hallucinations and apathy. Ultimately, the disease leads to a complete loss of self-care ability (Tzourio-Mazoyer et al., 2002). In 2011, the National Institute on Aging–Alzheimer's Association proposed new diagnostic guidelines for AD. These guidelines categorize AD into three stages: dementia due to AD, mild cognitive impairment (MCI) due to AD, and preclinical AD. The revised diagnostic criteria also incorporate structural, functional, and molecular imaging biomarkers (Vallières et al., 2015). Notably, both MCI and preclinical AD are considered early stages of CI and have a high risk of progressing to AD (Wu et al., 2018). Effective treatment for MCI and preclinical AD can help patients maintain their autonomy, stop disease progression, and improve future outcomes (Wu et al., 2019). Therefore, it is important to identify MCI as early as possible. Currently, the clinical diagnosis of MCI is mainly based on clinical history, psychiatric evaluation, and neurological examination, which is more subjective and may not accurately determine the stage of the disease accurately.

Magnetic resonance imaging (MRI) technology can non-invasively visualize the brain with high resolution. Due to its advantages of non-invasiveness, non-ionization, non-radiation, and affordable price, it has become the primary imaging diagnostic method for MCI diagnosis (Chandra et al., 2019). However, doctors have limited ability to identify small lesions based on visual images during MRI diagnosis. Radiomics was first proposed by Lambin in 2012 and is a new method that integrates medicine and computer science (Lambin et al., 2012). It can extract quantitative image features from standard images and reflect the heterogeneity of diseases through these features. Previous studies have demonstrated that MRI radiomics features of the entorhinal cortex and amygdala can accurately identify MCI at an early stage (Park et al., 2023; Betrouni et al., 2020; Wang et al., 2024; Du et al., 2023).

The role of the cerebellum in CI is an area of research that has received significant attention. Traditionally, the cerebellum was considered to be primarily involved in the regulation of motor function; however, a growing body of research suggests that the cerebellum also plays an important role in cognitive function. The cerebellum is closely connected to the cerebral cortex and affects cognitive functions such as motor coordination, learning, and memory. In recent years, researchers have conducted in-depth studies on the role of the cerebellum in CI using neuroimaging techniques. It has been found that cerebellar damage or abnormality is associated with some cognitive dysfunctions, such as learning disabilities, attention deficits, and memory disorders (Villemagne et al., 2013; Lin et al., 2024). It has also been shown that the cerebellum is involved in functions such as emotion regulation and social cognition (Hoche et al., 2018; Cheng et al., 2022; Rudolph et al., 2023). Increasingly, studies are being devoted to exploring the role of the cerebellum in CI. The development of radiomics has provided researchers with an additional tool to elucidate the role of the cerebellum in CI. Therefore, this study aimed to investigate whether cerebellar MRI-based radiomics features can identify CI, especially MCI.

## Methods

Data from patients with MCI, patients diagnosed with Alzheimer's disease (AD), and healthy controls (HCs) matched by gender and age were collected retrospectively from January 2022 to December 2023 at Fujian Medical University Union Hospital. Additionally, data from MCI and AD patients, collected from The First Affiliated Hospital of Xiamen University and The Third Hospital of Xiamen from January 2022 to December 2023, were used to validate the model for identifying MCI. All patients underwent brain MRI, which included T1, T2, and T2-FLAIR-weighted imaging. This study was reviewed and approved by the Ethics Committee of Union Hospital of Fujian Medical University (Approval No. 2025KY014).

Inclusion criteria: AD was diagnosed according to the National Institute of Neurological and Communicative Disorders and Stroke-Alzheimer's Disease and Related Disorders Association (NINCDS-ADRDA) criteria (Dubois et al., 2007). The clinical diagnostic criteria for MCI include (1) cognitive impairment reported by patients, informants, or experienced clinicians; (2) one or more cognitive impairments identified through neuropsychological tests; (3) slight impairment in performing complex instrumental daily activities, while continuing to manage independent daily living; and (4) not meeting the diagnostic criteria for AD.

Exclusion criteria were as follows: (1) poor image quality; (2) layer thickness >5 mm; (3) cognitive impairment due to various causes including vascular issues, inflammation, toxicity, pharmacogenetic factors, trauma, hydrocephalus, and tumors; comorbid severe psychiatric disorders (e.g., schizophrenia) or pre-existing psychiatric disorders that prevent patients from performing the related examinations as required; inability to complete the imaging examination due to claustrophobia or significant physical limitations; (4) history of carbon monoxide poisoning, chronic pain disorders, drug or alcohol dependency, or severe disabilities that hinder cooperation with the examination; (5) abnormal thyroid function, diabetes, severe heart failure, coronary heart disease and other heart-related diseases, renal failure, cirrhosis of the liver, history of malignant tumors, or other serious conditions that impede cooperation with the examination and treatment.

The T1, T2, and T2-FLAIR sequences were acquired on a GE Signa HDxt 1.5T scanner with the following parameters (T1: TR = 1,906.8 ms, TE =20.0 ms, slice thickness = 6 mm, 18 slices, flip angle =90°; T2: TR = 4,500 ms, TE =112 ms, slice thickness = 6 mm, 18 slices, flip angle =90°; T2-FLAIR: TR = 8,502 ms, TE =161 ms, slice thickness = 1 mm, 18 slices, flip angle =90°). For each volume of interest, a total of 57 radiomic features were extracted using the MATLAB-based image texture analysis toolkit (github.com/mvallieres/radiomics). These features can be divided into 2 groups: (1) 18 intensity features: these features quantify the density of the patch pair from the histogram of voxel intensities and (2) 39 texture features: textural features quantify the spatial arrangement of the patch pair by using the gray-level co-occurrence matrices (GLCM), gray-level run length matrices (GLRLM), gray-level size zone matrix (GLSZM), and neighborhood gray-tone difference matrix (NGTDM). The features are summarized in Table 1.

**Table 1:** The summary of 57 features.

**Feature category**	**Feature name**				**Feature number**
Intensity:					18
	Energy	h-Energy	Kurtosis	Max
	Mean absolute deviation	Mean	Media	Min
	Range	Root mean square	Skewness	Standard-deviation
	h-Uniformity	Variance	h-Mean	h-Variance
	h-Skewness	h-Kurtosis		
Texture:					39
GLCM	Energy	Contrast	Correlation	Homogeneity	
	Variance	Sun average	Entropy	Dissimilarity	
	Short run emphasis		Long run emphasis		
GLRLM	Gray-level nonuniformity		Run-length nonuniformity		
	Run percentage		Low gray-level run emphasis			
	High gray-level run emphasis		Short run low gray-level emphasis			
	Short run high gray-level emphasis		Long run low gray-level emphasis			
	Long run high gray-level emphasis		Gray-level variance			
	Run-length variance		Small zone emphasis			
	Large zone emphasis					
GLSZM	Gray-level nonuniformity		Zone-size nonuniformity		
	Zone percentage		Low gray-level zone emphasis		
	High gray-level zone emphasis		Small zone low gray-level emphasis		
	Small zone high gray-level emphasis		Large zone low gray-level emphasis		
	Large zone high gray-level emphasis		Gray-level variance		
	Zone-size variance				
NGTDM	Coarseness	Contrast	Busyness	Complexity	Strength	

The raw MRI images were first aligned with the standard brain space. The cerebellum was divided into 26 brain regions based on the AAL brain atlas; the name of each brain region is shown in Table 1, and the specific definitions of the brain regions were referred to Siddiqi et al. (2022). Finally, 18 grayscale and 39 texture features of each brain region image were extracted using the MATLAB-based image texture analysis toolkit (github.com/mvallieres/radiomics).

Radiological characteristics of each region of the cerebellum were extracted and screened based on each modal MRI T1, T2, and T2-FLAIR-weighted images. Based on the radiological features identified by feature screening, a sparse representation classifier based on non-parametric training is built for disease classification and diagnosis. The sparse representation classifier does not require parametric training of the model during classification, which simplifies the complexity of the model and can effectively inhibit model overfitting (Shu et al., 2020).

The data were divided into a 10-fold cross-validation set and an independent test set for model validation according to a ratio of 2:1. We first carried out 10-fold cross-validation on the cross-validation set. Then the entire cross-validation set was used as training data to train the final model for the test set.

Considering a binary classification problem, the training set has m+n samples, where m represents the number of samples in the first category and n represents the number of samples in the second category. The feature set of the training set samples can be expressed as $F=\left[f_{1}^{1},f_{2}^{1},\cdots \;,f_{m}^{1},f_{1}^{2},f_{2}^{2},\cdots \;,f_{n}^{2}\right]$, where the subscript represents the sample number and the superscript represents the class of the sample. The purpose of the classifier is to determine the class of the test sample feature *f* according to the training set sample feature *F*. For the sparse representation classifier, we first use the training set sample feature to sparsely represent the test sample feature *f*, that is, to solve the optimization problem of Equation 1.


(1) $$\begin{array}{l} \hat{\beta }=\underset{\beta }{\arg\min}||f-F\beta |{|}_{2}^{2}+\gamma {\left||\beta |\right|}_{0} \end{array}$$

where $\hat{\beta }$ is the sparse representation coefficient to be solved, γ is the sparse constraint control term, usually set to 0.01. Equation 1 can be solved by the orthogonal matching pursuit algorithm (Aharon et al., 2006). When the sparse representation coefficients are obtained, we calculate the reconstructed residuals *r*_*c*_(*f*) of each class by Equation 2:


(2) $$\begin{array}{l} r_{c}\left(f\right)=f-F\delta_{c}\left(\hat{\beta }\right),c=1,2 \end{array}$$

c represents the sample class, and δ_*c*_(·) represents setting all coefficients except the coefficient corresponding to the sample of the *c*-the class to zero. Finally, the residuals of each class are compared, and the class with the smallest residual is the class of the test sample:


(3) $$\begin{array}{l} \text{Id}\left(f\right)=\underset{c}{\arg\min}r_{c}\left(f\right) \end{array}$$

### Statistical methods

Statistical analyses were performed using the Statistical Package for the Social Sciences (SPSS, version 20.0, IBM). An independent samples' *t*-test or chi-square test was used to test statistical differences in characteristics between groups. Receiver operating characteristic (ROC) curve; area under curve (AUC); and sensitivity, specificity, positive predictive value, and negative predictive value were utilized to evaluate the performance of the model.

## Results

**Figure 1:**
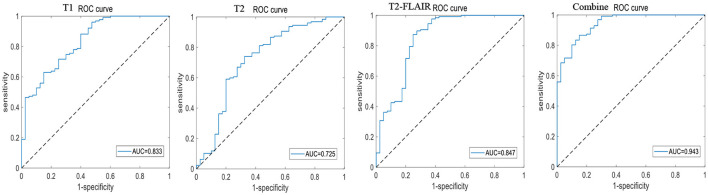
The ROC curve of training set based on cerebellar MRI radiomics for identifying CI.

**Figure 2:**
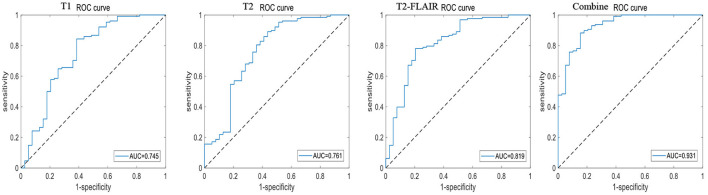
The ROC curve of training set based on hippocampal MRI radiomics for identifying CI.

**Figure 3:**
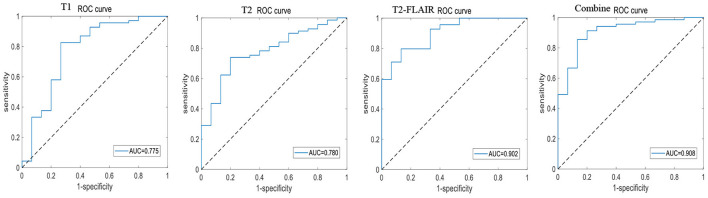
The ROC curve of independent test set based on cerebellar MRI radiomics for identifying CI.

**Figure 4:**
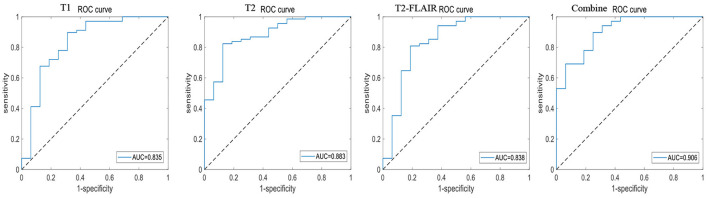
The ROC curve of independent test set based on hippocampal MRI radiomics for identifying CI.

A total of 196 patients with CI (CI group, 87 MCI and 109 AD) and 55 HCs (HC group) were included at the Fujian Medical University Union Hospital and divided into training and testing sets using a 10-fold cross-validation strategy in a 1:2 ratio. A total of 1,482 and 1,026 features were extracted from cerebellar and hippocampal MRI images, respectively. The accuracy, sensitivity, specificity, positive predictive value (PPV), and negative predictive value (NPV) of the 10-fold cross-training set for diagnosing CI in the cerebellar and hippocampal T1 modality, T2 modality, T2-FLAIR modality, and the combination of the three modalities are shown in Table 2, and the ROC curves are shown in Figures 1, 2. The accuracy, sensitivity, and specificity of the independent test set are shown in Table 3. The ROC curve is shown in Figures 3, 4. The model constructed by combining cerebellar MRI-radiomic features of three modalities is more effective in diagnosing CI than the model constructed by hippocampal MRI-radiomic features.

**Table 2:** The efficacy of training set based on cerebellar and hippocampal MRI radiomics for identifying CI.

**Sequence**	**Accuracy**	**Sensitivity**	**Specificity**	**Positive predictive value**	**Negative predictive value**
T1	0.850[0.847 0.852] vs. 0.820[0.818 0.823]	0.961[0.958 0.961] vs. 0.969[0.967 0.970]	0.500[0.491 0.505] vs. 0.333[0.322 0.338]	0.859[0.856 0.861] vs. 0.827[0.824 0.830]	0.800[0.788 0.802] vs. 0.765[0.749 0.770]
T2	0.802[0.799 0.804] vs. 0.832[0.828 0.834]	0.945[0.942 0.945] vs. 0.984[0.983 0.985]	0.350[0.342 0.356] vs. 0.333[0.325 0.338]	0.822[0.819 0.825] vs. 0.829[0.825 0.830]	0.667[0.647 0.669] vs. 0.867[0.860 0.877]
T2-Flair	0.886[0.886 0.890] vs. 0.850[0.847 0.852]	0.984[0.984 0.986] vs. 0.977[0.975 0.977]	0.575[0.570 0.585] vs. 0.436[0.428 0.442]	0.880[0.879 0.884] vs. 0.850[0.847 0.853]	0.920[0.916 0.927] vs. 0.850[0.839 0.854]
Combination	0.910[0.910 0.914] vs. 0.868[0.867 0.872]	0.992[0.991 0.993] vs. 1.000[1.000 1.000]	0.650[0.648 0.662] vs. 0.436[0.435 0.449]	0.900[0.900 0.904] vs. 0.853[0.852 0.857]	0.963[0.958 0.965] vs. 1.000[1.000 1.000]

[ ] indicates 95% confidence intervals.

**Table 3:** The efficacy of independent test set based on cerebellar and hippocampal MRI radiomics for identifying CI.

**Sequence**	**Accuracy**	**Sensitivity**	**Specificity**	**Positive predictive value**	**Negative predictive value**
T1	0.845 vs. 0.845	0.928 vs. 0.971	0.467 vs. 0.312	0.889 vs. 0.857	0.583 vs. 0.714
T2	0.810 vs. 0.869	0.913 vs. 1.000	0.333 vs. 0.312	0.863 vs. 0.861	0.455 vs. 1.000
T2-Flair	0.881 vs. 0.869	0.971 vs. 0.971	0.467 vs. 0.437	0.893 vs. 0.880	0.778 vs. 0.778
Combination	0.893 vs. 0.893	0.957 vs. 0.971	0.600 vs. 0.562	0.917 vs. 0.904	0.750 vs. 0.818

**Figure 5:**
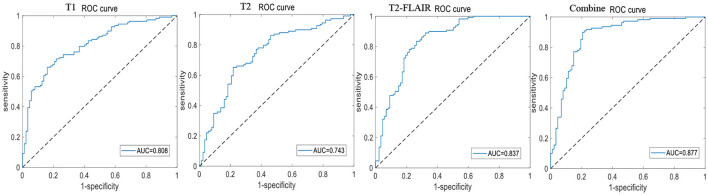
The ROC curve of training test set based on cerebellar MRI radiomics for identifying MCI.

**Figure 6:**
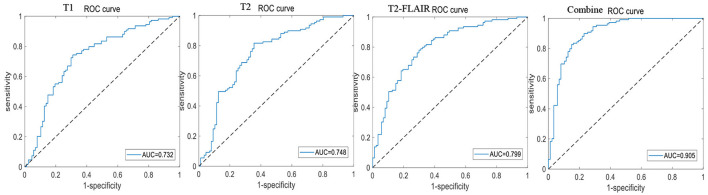
The ROC curve of training test set based on hippocampal MRI radiomics for identifying MCI.

**Figure 7:**
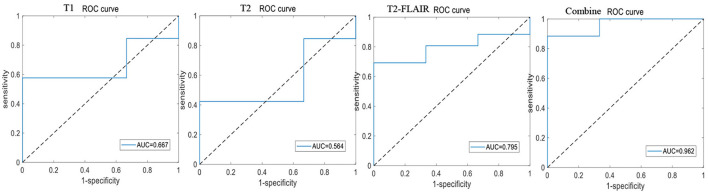
The ROC curve of cross external validation based on cerebellar MRI radiomics for identifying MCI.

**Figure 8:**
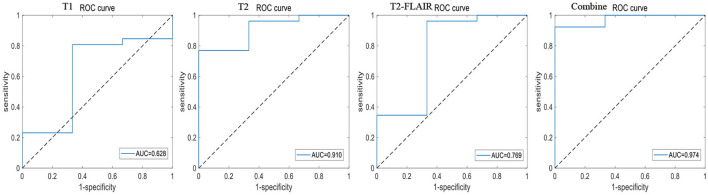
The ROC curve of cross external validation based on hippocampal MRI radiomics for identifying MCI.

Eighty-seven patients with MCI (MCI group) and 109 patients with AD (AD group), included from Fujian Medical University Union Hospital, were used to construct a model for identifying MCI. A total of 13 patients with MCI and 26 patients with AD, who were included from the other 2 hospitals, were used for further model external validation. The accuracy, sensitivity, specificity, positive predictive value (PPV) and negative predictive value (NPV) of the 10-fold cross-training set for diagnosing MCI in the cerebellar and hippocampal T1 modality, T2 modality, T2-FLAIR modality, and the combination of the three modalities are shown in Table 4. The ROC curves are shown in Figures 5, 6. The accuracy, sensitivity, and specificity of the independent test set are shown in Table 5. The ROC curve is shown in Figures 7, 8. The model constructed by combining cerebellar MRI-radiomics features of three modalities could diagnose MCI as accurately as the hippocampal.

**Table 4:** The efficacy of training set based on cerebellar and hippocampal MRI radiomics for identifying MCI.

**Sequence**	**Accuracy**	**Sensitivity**	**Specificity**	**Positive predictive value**	**Negative predictive value**
T1	0.735[0.731 0.737] vs. 0.714[0.713 0.718]	0.734[0.729 0.737] vs. 0.752[0.749 0.756]	0.736[0.730 0.739] vs. 0.667[0.665 0.674]	0.777[0.773 0.781] vs. 0.739[0.735 0.743]	0.688[0.682 0.690] vs. 0.682[0.681 0.689]
T2	0.709[0.706 0.712] vs. 0.735[0.729 0.735]	0.780[0.775 0.782] vs. 0.807[0.802 0.810]	0.621[0.616 0.627] vs. 0.644[0.636 0.645]	0.720[0.718 0.725] vs. 0.739[0.731 0.738]	0.692[0.686 0.695] vs. 0.727[0.723 0.733]
T2-Flair	0.791[0.789 0.794] vs. 0.745[0.742 0.747]	0.881[0.878 0.883] vs. 0.844[0.840 0.845]	0.678[0.676 0.685] vs. 0.621[0.617 0.626]	0.774[0.770 0.777] vs. 0.736[0.732 0.739]	0.819[0.817 0.825] vs. 0.761[0.755 0.763]
Combination	0.852[0.848 0.853] vs. 0.837[0.832 0.837]	0.917[0.913 0.918] vs. 0.945[0.941 0.944]	0.770[0.765 0.773] vs. 0.701[0.696 0.705]	0.833[0.829 0.835] vs. 0.798[0.794 0.800]	0.882[0.877 0.883] vs. 0.910[0.904 0.910]

[ ] indicates 95% confidence intervals.

**Table 5:** The efficacy of cross external validation based on cerebellar and hippocampal MRI radiomics for identifying MCI.

**Sequence**	**Accuracy**	**Sensitivity**	**Specificity**	**Positive predictive value**	**Negative predictive value**
T1	0.724 vs. 0.793	0.769 vs. 0.885	0.333 vs. 0.000	0.909 vs. 0.885	0.143 vs. 0.000
T2	0.793 vs. 0.828	0.885 vs. 0.846	0.000 vs. 0.667	0.885 vs. 0.957	0.000 vs. 0.333
T2-Flair	0.793 vs. 0.828	0.846 vs. 0.846	0.333 vs. 0.667	0.917 vs. 0.957	0.200 vs. 0.333
Combination	0.897 vs. 0.931	0.923 vs. 0.962	0.667 vs. 0.667	0.960 vs. 0.962	0.500 vs. 0.667

## Discussion

This study found that the diagnostic accuracy of CI based on the three modalities of cerebellar T1, T2, and T2-FLAIR imaging radiomic features was all over 80.0%, with a sensitivity of 95.0% or higher, but poor specificity. However, the combined diagnostic accuracy of the three modalities imaging radiomic features for the CI training set reached 91.0%, with a sensitivity of 99.2% and an improved specificity of 65.0%, even a bit better than hippocampal. The area under the ROC curve was 0.943, and the accuracy of the independent test set was also 89.3%, with a sensitivity of 95.7% and a specificity of 60.0%. The area under the ROC curve was 0.908. This study further demonstrated that the combined diagnostic accuracy of cerebellar T1-, T2-, and T2-FLAIR modality radiomic features for MCI reached 85.2%, sensitivity was 91.7%, specificity was 77%, ROC curve area under was 0.877, similar to hippocampal and the model accuracy of external validation reached to 89.7%, sensitivity was 92.3%, specificity was 66.7%, and the ROC curve area under was 0.962. The results have significant clinical value in improving the ability of MRI to identify MCI, providing necessary theoretical and practical support for the early identification, intervention, and treatment of CI.

Radiomics is a new method that integrates medicine and computer science. It can reflect the heterogeneity of diseases through image features and is characterized by low cost and non-invasiveness. In traditional clinical diagnosis, doctors rely on the visual interpretation of images and have a limited ability to recognize small lesions. Radiomics can provide a more accurate and objective basis for qualitative and quantitative analysis of diseases. MRI radiomics have been applied in the diagnosis and prediction of MCI, and there have been many results in this area of research (Song et al., 2024; Yan et al., 2024). MRI radiomic features of the entorhinal cortex and amygdala can accurately identify MCI early. Whether MRI radiomic features of the cerebellum can accurately identify MCI is not clear.

Patients collected from three tertiary hospitals in this study underwent MRI imaging acquisition, radiomics feature extraction, feature screening, and classification model building in accordance with a standardized process to ensure standardization and uniformity of data quality. Meanwhile, the study utilizes a sparse representation-based method to select a few high-resolution features for subsequent classification. The sparse representation classifier requires no parameter training for the model during classification, thereby simplifying the complexity of the model and effectively inhibiting model overfitting. In addition, the study conducted external validation after establishing the diagnostic model.

When there is a lesion in the brain tissue, the texture of MR images may change accordingly. Leandrou et al. (2020) extracted texture features from normal controls (NC), MCI, MCI that progressed to AD (MCI converters, MCIc), and AD subjects and found statistically significant differences in the textural features of the entorhinal cortex. The AUC was 0.710 for NC, 0.730 for MCI and MCIc, and 0.764 for MCI and AD, which indicated that entorhinal cortical textural features could classify and diagnose MCI. Zheng et al. (2022)achieved an accuracy of 72.5% in classifying AD and MCI based on hippocampal texture, and the accuracy for MCI and NC classification was also 72.5%. MCI and NC were classified with an accuracy of 75%. Sivaranjini and Sujatha (2021) applied amygdala texture characterization and found that AD was classified as amnestic mild cognitive impairment (aMCI) with an accuracy of 0.81 and an AUC of 0.84, while aMCI was classified as NC with an accuracy of 0.75 and an AUC of 0.80. This study identified the value of cerebellar MRI imaging histology in diagnosing MCI and classifying AD and MCI. These results indicate that MRI texture analysis can quantitatively characterize tissues, reflect their physiological and pathological stages, and reveal information in the image that is not recognizable by the naked eye.

The cerebellum has been thought to be associated with motor control, and few studies have been conducted on the diagnosis of CI and classification of AD and MCI based on cerebellar MRI imaging histology. In recent years, with advances in neuroimaging and neuropathological, the role of the cerebellum in cognitive dysfunction has gradually been redefined. This study revealed that the value of cerebellar-based multimodal MRI radiomic features in the diagnosis of MCI and the classification of AD and MCI is not inferior to, and may even be superior to, that of the internal olfactory cortex, hippocampus, and amygdala, which provides an important imaging biomarker for the diagnosis of MCI. In 2023, Smith (2023) demonstrated that the dentato-thalamo-cortical pathway facilitates bidirectional communication between the cerebellum and the prefrontal cortex. Disruption of this circuit is correlated with deficits in executive function and working memory, as evidenced by functional MRI (fMRI) studies showing cerebellar-default mode network (DMN) decoupling preceding hippocampal atrophy in early AD. Cryo-EM structural analyses reveal that cerebellar Purkinje cells internalize tau fibrils originating from temporoparietal cortices via prion-like mechanisms, triggering synaptic loss in the granular layer (Parra Bravo et al., 2024). Single-nucleus RNA sequencing reveals a 40% reduction in mitochondrial complex IV activity in cerebellar neurons during MCI, preceding hippocampal alterations (Bakooshli et al., 2023). These are possible pathophysiological mechanisms of cerebellar involvement in CI.

This study has several limitations. First, all patients were included from different tertiary hospitals in Fujian. The representative sample may be insufficient, thereby limiting the generalizability of the findings to local memory clinics and primary care. Second, the sample size used for validation needs to be expanded to verify the versatility of the model we created. Third, this study evaluates the diagnostic value of multimodal magnetic resonance imaging of the cerebellum without the use of biomarkers in combination with clinical symptoms.

In conclusion, this study has shown that multimodal magnetic resonance imaging of the cerebellum has significant and high diagnostic value in MCI in China. These findings may provide a reference and direction for early diagnosis and intervention of MCI. Future studies employing large-scale multicenter prospective clinical protocols are needed to further clarify the impact of multimodal magnetic resonance imaging of the cerebellum in the Chinese population. Additional economic data are required to be combined to assess the cost-effectiveness of multimodal magnetic resonance imaging of the cerebellum in clinical routine.

## Data Availability

The raw data supporting the conclusions of this article will be made available by the authors, without undue reservation.
